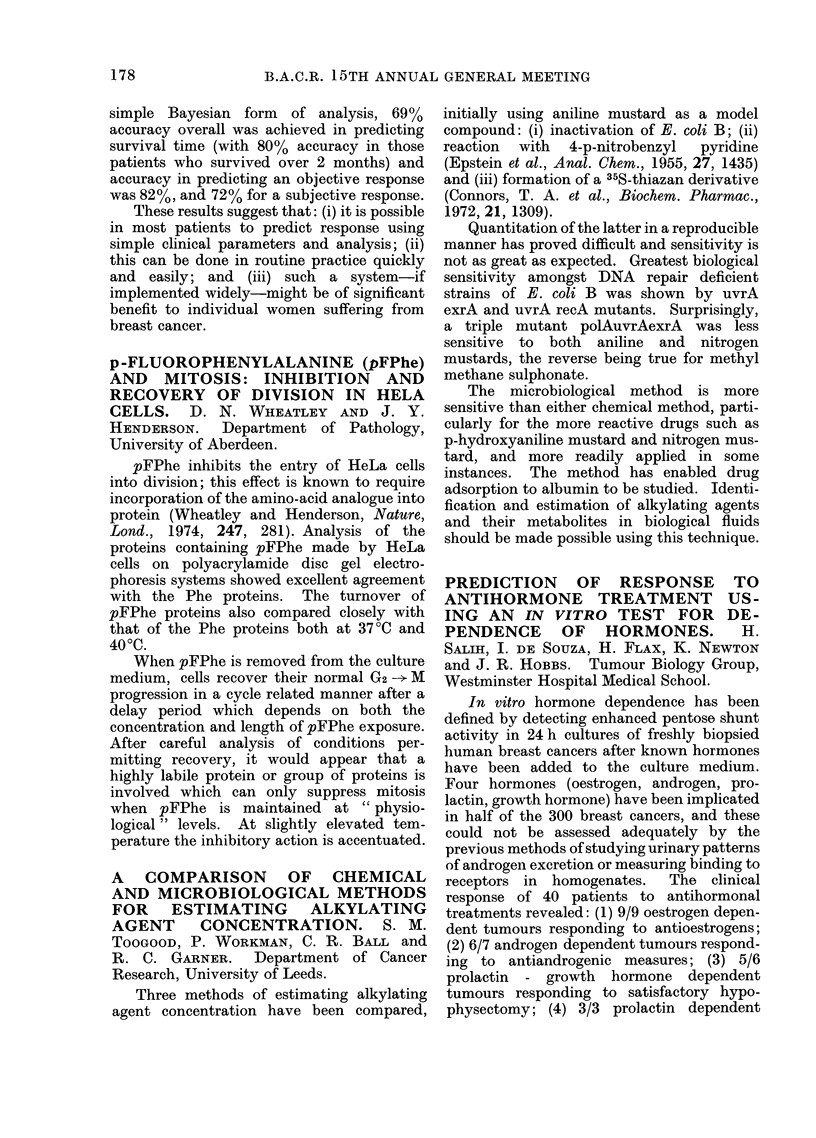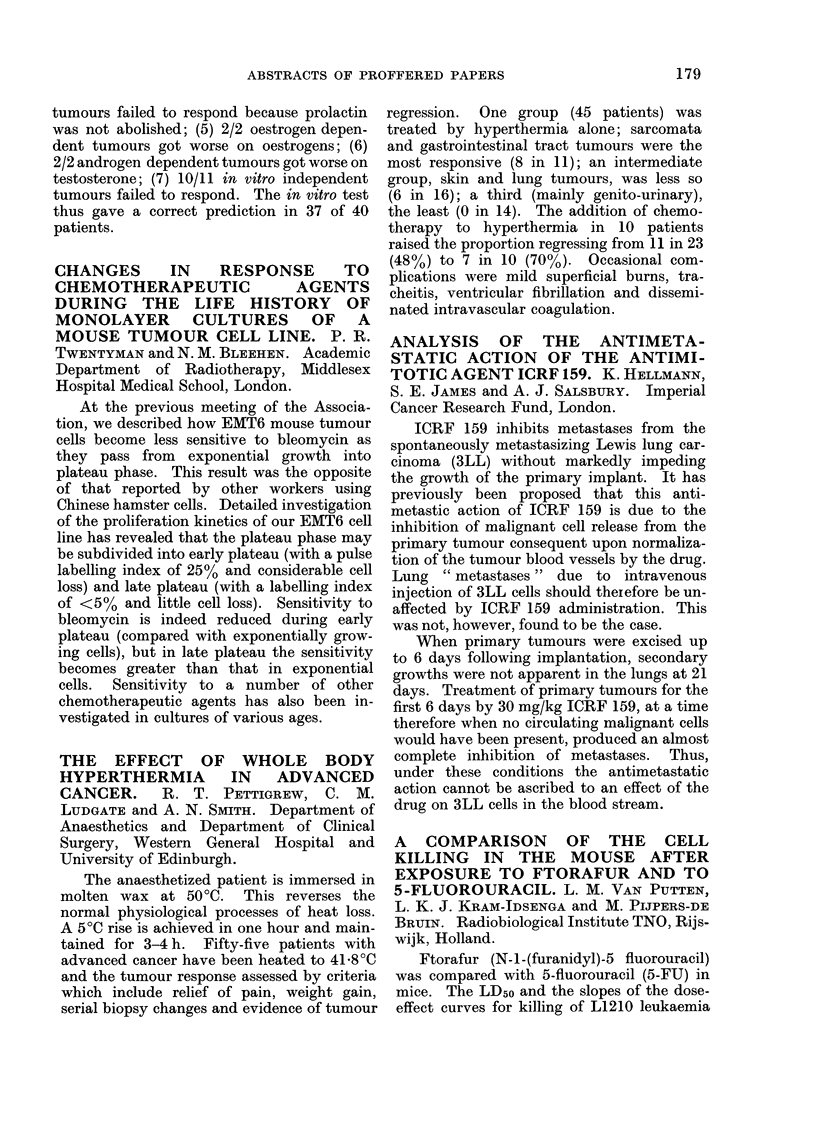# Proceedings: Prediction of response to antihormone treatment using an in vitro test for dependence of hormones.

**DOI:** 10.1038/bjc.1974.150

**Published:** 1974-08

**Authors:** H. Salih, I. de Souza, H. Flax, K. Newton, J. R. Hobbs


### PREDICTION OF RESPONSE TO ANTIHORMONE TREATMENT USING AN *IN VITRO* TEST FOR DEPENDENCE OF HORMONES.

H. SALIH, I. DE SOUZA, H. FLAX, K. NEWTON and J. R. HOBBS. Tumour Biology Group, Westminster Hospital Medical School.

*In vitro* hormone dependence has been defined by detecting enhanced pentose shunt activity in 24 h cultures of freshly biopsied human breast cancers after known hormones have been added to the culture medium. Four hormones (oestrogen, androgen, prolactin, growth hormone) have been implicated in half of the 300 breast cancers, and these could not be assessed adequately by the previous methods of studying urinary patterns of androgen excretion or measuring binding to receptors in homogenates. The clinical response of 40 patients to antihormonal treatments revealed: (1) 9/9 oestrogen dependent tumours responding to antioestrogens; (2) 6/7 androgen dependent tumours responding to antiandrogenic measures; (3) 5/6 prolactin - growth hormone dependent tumours responding to satisfactory hypophysectomy; (4) 3/3 prolactin dependent tumours failed to respond because prolactin was not abolished; (5) 2/2 oestrogen dependent tumours got worse on oestrogens; (6) 2/2 androgen dependent tumours got worse on testosterone; (7) 10/11 *in vitro* independent tumours failed to respond. The *in vitro* test thus gave a correct prediction in 37 of 40 patients.